# Mechanical versus humoral determinants of brain death-induced lung injury

**DOI:** 10.1371/journal.pone.0181899

**Published:** 2017-07-28

**Authors:** Asmae Belhaj, Laurence Dewachter, Sandrine Rorive, Myriam Remmelink, Birgit Weynand, Christian Melot, Emeline Hupkens, Céline Dewachter, Jacques Creteur, Kathleen Mc Entee, Robert Naeije, Benoît Rondelet

**Affiliations:** 1 Department of Cardio-Vascular, Thoracic Surgery and Lung Transplantation, CHU UcL Namur, Université Catholique de Louvain, Yvoir, Belgium; 2 Laboratory of Physiology and Pharmacology, Faculty of Medicine, Université Libre de Bruxelles, Brussels, Belgium; 3 Department of Anatomopathology, Erasmus Academic Hospital, Brussels, Belgium; 4 DIAPATH—Center for Microscopy and Molecular Imaging (CMMI), Gosselies, Belgium; 5 Department of Anatomopathology, UZ Leuven, Katholiek Universiteit Leuven, Brussels, Belgium; 6 Department of Emergency, Erasmus Academic Hospital, Brussels, Belgium; 7 Department of Intensive Care, Erasmus Academic Hospital, Brussels, Belgium; National Institutes of Health, UNITED STATES

## Abstract

**Background:**

The mechanisms of brain death (BD)-induced lung injury remain incompletely understood, as uncertainties persist about time-course and relative importance of mechanical and humoral perturbations.

**Methods:**

Brain death was induced by slow intracranial blood infusion in anesthetized pigs after randomization to placebo (n = 11) or to methylprednisolone (n = 8) to inhibit the expression of pro-inflammatory mediators. Pulmonary artery pressure (PAP), wedged PAP (PAWP), pulmonary vascular resistance (PVR) and effective pulmonary capillary pressure (PCP) were measured 1 and 5 hours after Cushing reflex. Lung tissue was sampled to determine gene expressions of cytokines and oxidative stress molecules, and pathologically score lung injury.

**Results:**

Intracranial hypertension caused a transient increase in blood pressure followed, after brain death was diagnosed, by persistent increases in PAP, PCP and the venous component of PVR, while PAWP did not change. Arterial PO_2_/fraction of inspired O_2_ (PaO_2_/FiO_2_) decreased. Brain death was associated with an accumulation of neutrophils and an increased apoptotic rate in lung tissue together with increased pro-inflammatory interleukin (IL)-6/IL-10 ratio and increased heme oxygenase(HO)-1 and hypoxia inducible factor(HIF)-1 alpha expression. Blood expressions of IL-6 and IL-1β were also increased. Methylprednisolone pre-treatment was associated with a blunting of increased PCP and PVR venous component, which returned to baseline 5 hours after BD, and partially corrected lung tissue biological perturbations. PaO_2_/FiO_2_ was inversely correlated to PCP and lung injury score.

**Conclusions:**

Brain death-induced lung injury may be best explained by an initial excessive increase in pulmonary capillary pressure with increased pulmonary venous resistance, and was associated with lung activation of inflammatory apoptotic processes which were partially prevented by methylprednisolone.

## Introduction

Brain death (BD) induced lung injury also called neurogenic pulmonary edema (NPE) is an acute condition which follows closely a variety of insults to the central nervous system that cause acute, rapid, abrupt and extreme increase in intracranial pressure (ICP) [1–3]. The pathophysiology linking the neurologic, cardiac, and pulmonary conditions in NPE remains debated.

The abrupt increase in ICP leading to neuronal compression, ischemia or damage is believed to give rise to an intense activation of the sympathetic nervous system and the release of catecholamines [4].

The autonomic response to elevated ICP plays an important role in the pathogenesis of NPE, however, what occurs mechanistically at the level of the pulmonary vascular endothelium remains uncertain and theoretical. Several clinicopathologic paradigms have been proposed to explain the clinical syndrome of NPE.

In the neuro-cardiac theory, the catecholamines storm following the neurologic insult leads to direct toxic myocardial injury as described in the Takotsubo’s cardiomyopathy and the development of pulmonary edema [5].

The neuro-hemodynamic and the “blast” posit that the severe abrupt increases in systemic and pulmonary pressures following the catecholamine surge result in a net shift of blood volume from the systemic circulation to the low resistance pulmonary circulation. This increase in pulmonary venous pressure leads to the development of transudative pulmonary edema [6]. The “blast theory” further posits that the acute rise in capillary pressure induces a degree of barotrauma capable of damaging the capillary-alveolar membrane [3]. And the “pulmonary venule adrenergic hypersensitivity” hypothesis is that the massive sympathetic discharge following ICP directly affects the pulmonary vascular bed with pulmonary venoconstriction or endothelial disruption responsible for the formation of pulmonary edema [2].

Moreover, the early autonomous storm accompanying brain death triggers the development of a systemic and pulmonary inflammatory response, which may lead, by itself, to increased pulmonary endothelial permeability [7,8] and sympathetic vasoconstriction of systemic and pulmonary vessels.

Finally, besides this initial neurogenic pulmonary edema development, occurrence of severe cardiac dysfunction and/or large vascular filling could aggravate lung function alteration by conjunction of a hydrostatic component.

A better characterization and quantification of a single “primum movens” of brain death induced lung injury would be of therapeutic relevance, and could also help to improve the survival of transplanted brain-death donor lungs [7,9].

We therefore investigated the biology and the pathophysiology of lung injury in a pig model of brain death [10]. Pulmonary capillary pressure (PCP) as a determinant of hydrostatic lung edema was determined from the analysis of pressure decay curves after single pulmonary arterial occlusion [11]. Lung tissue was sampled to quantify gene expressions and, when possible, protein levels of pro- and anti-inflammatory mediators thought to be involved in prolonged brain death induced lung injury as a permeability lung lesion [12], i.e. a SDRA variant [13]. The experiments were performed with and without preventive administration of methylprednisolone to blunt the humoral participation to brain death induced lung injury pathophysiology [14]. High-dose corticosteroids are standard practice in lung transplantation and have indeed been shown to improve the quality of brain-death donor lungs, presumably through non-specific anti-inflammatory effects [15].

## Materials and methods

The present study was approved by Institutional Ethics Committee on Animal Welfare at the Faculty of Medicine of the *Université Libre de Bruxelles* (Brussels, Belgium; protocol number: 510N) and was done in accordance with the “Guiding Principles in Care and Use of Animals” of the American Physiologic Society. The animals were delivered the day before the experiments by an approved breeder (Jan Derook Farm, Belgium) and have been placed under a water diet for six hours before anesthesia.

### Animal preparation

Female pigs (aged 4–5 months), weighting 43 ± 1 kg, were premedicated with intramuscular 20 mg.kg^-1^ ketamine and 0.1 mmg.kg^-1^ midazolam, anesthetized with intravenous 1 mg.kg^-1^ midazolam and 15 mg.kg^-1^ remifentanil and paralyzed for thoracotomy with intravenous 0.2 mg.kg^-1^.hour^-1^ rocuronium bromide. Anaesthesia was maintained during the protocol with intravenous 0.1 mg.kg^-1^.hour^-1^ midazolam and 7.5 mg.kg^-1^.hour^-1^ remifentanil. Animals were ventilated with an inspired oxygen fraction (FiO_2_) of 0.4 to 1.0 to maintain arterial oxygen saturation (SaO_2_) > 90%, a respiratory rate of 12–20 breaths.min^-1^ and a tidal volume of 15–25 mL.kg^-1^ to achieve an arterial PaCO_2_ between 35 and 45 mmHg, and a positive end-expiratory pressure (PEEP) between 5 and 8 cm H_2_O [9].

A flow-directed balloon-tipped pulmonary artery catheter (131H-7F; Baxter-Edwards, Irvine, CA, USA) was inserted via the left external jugular vein and positioned in the pulmonary artery for measurements of pulmonary artery pressure (PAP), wedged PAP (PAWP), right atrial pressure (RAP), PCP computed from the PAP decay curve after arterial balloon occlusion, thermodilution cardiac output (CO), and central body temperature, and for mixed venous blood sampling. A polyethylene catheter was inserted in the thoracic aorta via the right carotid artery to measure systemic artery pressure (SAP) and to sample arterial blood. Balanced crystalloid and gelatin modified solutions were perfused (at 10 mL.kg^-1^.hour^—1^) to maintain RAP between 6 and 8 mmHg. When the SAP < 65mmHg, infusion of norepinephrine was started to maintain blood pressure.

### Brain death procedure

The pigs were randomly assigned to an intravenous administration of placebo (BD group; n = 11) or 15 mg.kg^-1^ methylprednisolone sodium succinate (Solumedrol®, Pfizer, Ireland; BD + corticosteroids group; n = 8). A hole was drilled in the temporo-parietal region of the cranium. Slow parenchymal infusion (1.5 mL/min; 150 min) of autologous blood was performed through an 18-gauge SURFLO® catheter (TERUMO®, SR-OX1851CA) subdurally inserted to induce brain death and to record intracranial pressure (B). One hour after Cushing reflex response, i.e. a sharp increase in SAP with decreased HR [16], which lasted between 30 and 60 minutes, brain death was objectivated following standard criteria [17,18]: a) deep coma, excluding reversible factors such as anesthesia and hypothermia; b) absence of oculopupillary and corneal reflexes on two occasions; c) loss of spontaneous breathing with positive apnea testing; and d) no increase in HR after 1 mg of atropine.

Anesthesia and paralysis were withheld when the Cushing reflex was observed. After objectivation of brain death, protective ventilation was applied with tidal volume of 6 mL/kg and target plateau pressures <30 cmH_2_O and periodic deep inspirations were administered to prevent atelectasis.

### Hemodynamic measurements

A first data set was obtained at baseline after 20 min postoperative stabilization as assessed by stable heart rate (HR), SAP, PAP and CO. A second and a third data set were obtained respectively 1 hour and 5 hours after the Cushing reflex response, and defined as BD + 1h and BD + 5h. Finally, the animals were euthanized by pentobarbital sodium (Nembutal®, Oak, USA) overdose and lung tissue was sampled, immediately snap-frozen and stored at –80°C for pathobiological evaluation and, after overnight fixation, embedded in paraffin for immunohistochemistry.

Each data set included blood gas analysis and acquisition of SAP, PAP, PAWP, RAP, PCP and CO. After the acquisition of each data set, systemic arterial plasma was collected and lung biopsied using Endo-GIA™ 30mm Reload (Covidien, Ireland) in the upper left pulmonary lobe.

PCP and resulting venous compartmental resistance (Rv) was computed in triplicate by a dual-exponential fitting of the pressure decay curve obtained after pulmonary arterial balloon occlusion, as previously reported [11,19].

### Biological and histological assessments

#### Real-time quantitative polymerase chain reaction (RTQ-PCR)

Total RNA was extracted from snap-frozen lung tissue using the QIAGEN RNeasy® Mini kit (QIAGEN, Hilden, Germany), according to the manufacturer’s instructions. Total RNA content was determined by standard spectrophotometric techniques and RNA integrity was assessed by visual inspection of Gel Red (Biofilm, Hayward, CA)-stained agarose gels. Reverse transcription was performed using random primers and Superscript^TM^ II Reverse Transcriptase (Invitrogen, Carlsbad, CA, USA), according to the manufacturer’s instructions.

For RTQ-PCR, sense and antisense primers were designed using Primer3 program for *sus scrofa* tumor necrosis factor (TNF)-α, interleukin(IL)-6, IL-10, IL-1beta (IL-1β), IL-8, hypoxia inducible factor-1-alpha (HIF-1α), heme oxygenase-1 (HO-1), glutathion peroxidase-1 (GPx-1), oxidative-stress responsive-1 (OXSR-1), intercellular adhesion molecule (ICAM)-1, vascular cell adhesion protein (VCAM)-1, Bax, Bcl-2 and hypoxanthine phosphoribosyl transferase (HPRT)-1, β-actin (ACTB), TATA box binding protein (TBP1) and ribosomal protein L4 (RPL4), used as housekeeping genes as previously reported [20] (Table 1). To avoid inappropriate amplification of residual genomic DNA, intron-spanning primers were selected when exon sequences were known and analysis was run to check if primer pairs were selected when exon sequences were known and analysis was run to check if primer pairs were only matching the sequence of interest. For each sample, amplification reaction was performed in triplicate using SYBR-Green PCR Master Mix (Quanta Biosciences, Gaithersburg, MD, USA), specific primers and diluted template cDNA. Result analysis was performed using iCycler System (Bio-Rad Laboratories). Relative mRNA quantification was achieved using the Pfaffl method [21] by normalization with the housekeeping genes.

**Table 1:** Primers used for RTQ-PCR in porcine lung tissue.

Genes		Oligo sequences
**IL-6**	F	CCACCAGGAACGAAAGAGAG
*Interleukin-6*	R	AGTAGCCATCACCAGAAGCAG
**IL-10**	F	TCATCAATTTCTGCCCTGTG
*Interleukin-10*	R	TGTAGACACCCCTCTCTTGGA
**IL-1β**	F	CACCCAAAACCTGGACCTT
*Interleukin-1β*	R	TCTGCCTGATGCTCTTGTTC
**IL-8** *Interleukin-8*	F R	AGCTGGAAATCCTGTTTTGG TGAGGTGCAGTTGAGCAGAG
**ICAM-1**	F	ATTGTGAGGGGTGTCGAAGT
*Intercellular adhesion molecule-1*	R	TTCCCAGTTGTGTGTTTCCA
**VCAM-1**	F	TCCTCGTCACACAGCAACTA
*Vascular cell adhesion molecule-1*	R	GAGAAACGGCAAACACCATC
**TNF-α**	F	TCTGGACTTTGCTGAATCTGG
*Tumor necrosis factor-α*	R	TGAGGGGGTCTGAAGGAGTAA
**Bax**	F	CGCATTGGAGATGAACTGG
*Bcl2 associated X protein*	R	CGCCACTCGGAAAAAGACT
**Bcl2**	F	GACTTTGCCGAGATGTCCAG
*B-cell Lymphoma-2*	R	ACAATCCTCCCCCAGTTCA
**HO-1**	F	ATGTGAATGCAACCCTGTGA
*Heme Oxygenase-1*	R	GGAAGCCAGTCAAGAGACCA
**HIF-α**	F	TCCACCCAGGACACTGATTT
*Hypoxia inducible factor-1-alpha subunit*	R	TGGTGACAACTGATCGAAGG
**GPx-1**	F	TGGGGAGATCCTGAATTG
*Glutathion peroxidase-1*	R	GATAAACTTGGGGTCGGT
**OXSR-1**	F	AATAGGATTTGCCCAGCTCA
*Oxidative-stress responsive-1*	R	GTGGGTTTAGGCCAACAGAA
**HPRT**	F	TCAAGCAGCATAATCCAAAGATGG
*Hypoxanthine guanine phosphoribosyl transferase*	R	TCAAATCCAACAAAGTCTGGTC
**ACTB**	F	CACGCCATCCTGCGTCTGGA
*Actin beta*	R	AGCACCGTGTTGGCGTAGAG
**RPL4**	F	CAAGAGTAACTACAACCTTC
*Ribosomal protein-L4*	R	GAACTCTACGATGAATCTTC
**TBP1**	F	AACAGTTCAGTAGTTATGAGCCAGA
*TATA-box-binding protein-1*	R	AGATGTTCTCAAACGCTTCG

#### Protein extraction and Enzyme-linked immunosorbent assay (ELISA)

Proteins were extracted from snap-frozen lung tissue by homogenization in an appropriate ice-cold lysis buffer. After centrifugation, protein concentration was determined using the Bradford method with Bio-Rad protein Assay (Bio-Rad, Temse, Belgium). Protein levels of IL-6, IL-10 and IL-1β were determined with Multiplex Assay (MILLIPLEX^MAP^ Porcine Cytokine/Chemokine Magnetic Bead Panel, Merck Millipore, Darmstadt, Germany) in total lung homogenates and in systemic arterial serum, according to manufacturer’s instructions. Cytokine concentrations were obtained by referring to a standard curve realized in parallel. Results represented the mean value of two separate measurements performed in duplicate.

#### Immunohistochemistry–Lung evaluation of inflammatory cell infiltrates

Immunohistochemistry were performed as previously described [10]. After progressive rehydration, 5μm-sections of lung tissue fixed and embedded in paraffin were incubated in target retrieval buffer (Dako, Glostrup, Denmark) and heated in a bath for 20 minutes at 95°C for target retrieval. Endogenous peroxidase activity was quenched with hydrogen peroxide in phosphate buffered saline (PBS; 5%) for 10 minutes, and the sections were blocked by incubation with bovine serum albumin in PBS (3%) for 30 minutes. Sections were allowed to react for 60 minutes at 37°C with polyclonal anti-Pig myeloperoxidase (MPO) (Abcam, Cambridge, UK) antibodies diluted at 1/100 in PBS. Sections were then incubated with biotinylated anti-rabbit IgG at 1/200 (Vector Laboratories, Burlingame, U.S.A.) and subsequently with streptavidin–peroxidase (Dako). Antibody binding was detected with a liquid DAB (diaminobenzidine) substrate kit (AbCys Biology, Paris, France). The appearance of a brown reaction product was observed under a light microscope. Tissues were counterstained with hematoxylin (Sigma-Aldrich, St Louis, MO). Negative controls run without the primary antibody were tested and were found to be negative.

In each lung tissue slide and for each staining, twenty microscopic fields (light microscopy, X400 magnification) were randomly chosen.

The number of extravascular neutrophils (MPO-positive) was counted. Intravascular inflammatory cells were excluded. The total surface of each sample was then measured using ImageJ software. The average numbers of extravascular inflammatory cells per mm^2^ were then calculated as the total score for each specimen. Two investigators, who were blinded to the assigned group, did all cell counts independently. The mean value was used for analysis.

#### Immunohistochemistry—Terminal deoxynucleotidyl transferase dUTP nick-end labelling (TUNEL)

Lung epithelial cells undergoing apoptosis were detected by TUNEL staining using the ApopTag® Plus Peroxidase In Situ Apoptosis Detection Kit (EMD Millipore, Temecula, CA, USA), as previously described [22]. Negative control run without TdT enzyme and positive control pre-treated with DNase were tested. For each tissue specimen, twenty randomly chosen fields were examined. Lung epithelial apoptotic rate was calculated as the ratio of apoptotic cells (TUNEL-positive or brown nuclei) to total cells (brown + blue nuclei) (x100 to be expressed in percentage). Two independent investigators performed all counts in a blinded manner. The mean value was used for analysis.

#### Lung injury scoring

The lung injury was scored as previously reported [23]. Hematoxylin and eosin-stained sections were analyzed for neutrophil infiltration, airway epithelial-cell damage, interstitial edema, hyaline membrane formation, hemorrhage, and total lung injury score as the sum of those criterion. Each criterion was scored on a scale of 0 to 4, where 0 = normal, 1 = minimal change, 2 = mild change, 3 = moderate change, and 4 = severe change. Two independent investigators performed all counts in duplicate in a blinded manner.

### Statistical analysis

All data were expressed as mean +/- SEM (standard error of the mean). The data were analyzed using a 2 factors analysis of variance (group x time) with repeated measures on time and interaction group x time. When the F ration reached a significant critical value (p < 0.05) a Fischer’s protected t-test was performed with a Bonferroni’s correction for multiple comparisons.

Using univariable and multivariable analyses with the Cox proportional hazard model, we tested the associations between the hemodynamic and inflammatory variables and the occurrence of PaO_2_/FiO_2_ ratio impairment and the occurrence of histological lung injury assessed by ALI score. Value of p<0.05 was considered statistically significant.

## Results

Intracranial blood infusion was associated with an increase in intra-cranial pressure above 80 mmHg in 5 to 10 min and maintained subsequently higher than SAP. Adjustments were required during Cushing reflex-related hypertension that lasted 30 to 60 min.

### Hemodynamic evaluation

#### Brain death

**Fig 1:**
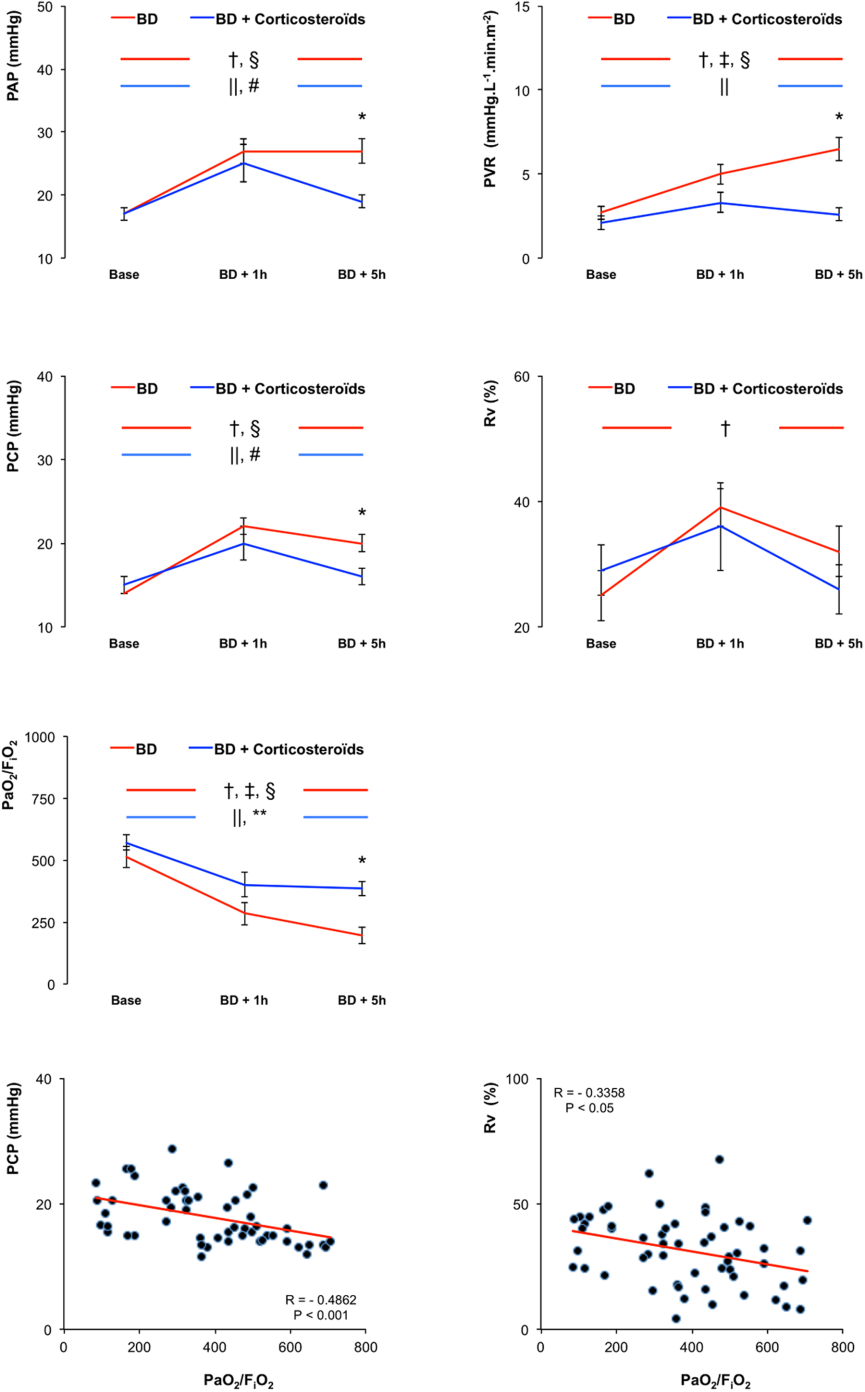
Hemodynamic evaluation. Mean pulmonary artery pressure (PAP), pulmonary vascular resistance (PVR), pulmonary capillary pressure (PCP), venous compartmental resistance (Rv), arterial PO_2_ divided by the fraction of inspired O_2_ (PaO_2_/FiO_2_) in pigs 1 hour and 5 hours after brain death (BD), with placebo-pretreatment (BD group, in red) or with preventive methylprednisolone (BD + Corticosteroids group, in blue) and correlation between PCP or Rv and arterial PO_2_ divided by the fraction of inspired O_2_ (PaO_2_/FiO_2_). Values are expressed as mean ± SEM. * p<0.05 BD versus BD + Corticosteroids; † p<0.05 Base versus BD + 1 hour, ‡ p<0.05 BD + 1 hour versus BD + 5 hours, § p<0.05 Base versus BD + 5 hours in the BD group; || p<0.05 Base versus BD + 1 hour, # p<0.05 BD + 1 hour versus BD + 5 hours, ** p<0.05 Base versus BD + 5 hours in the BD + Corticosteroids group.

**Fig 2:**
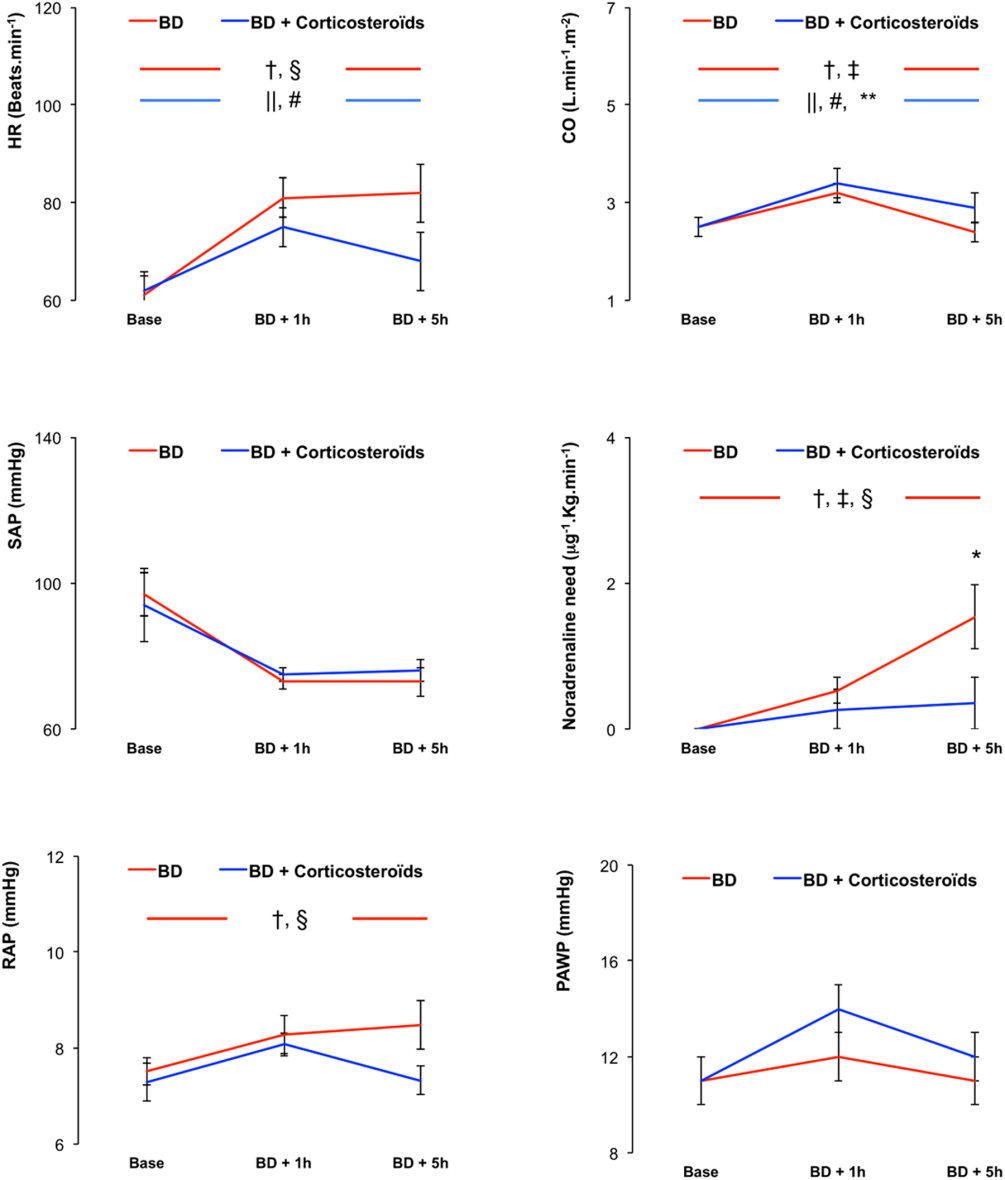
Hemodynamic evaluation. Heart rate (HR), Cardiac output (CO), Mean systemic artery pressure (SAP), Noradrenaline need, Right atrial pressure (RAP), Pulmonary artery wedge pressure (PAWP) in pigs 1 hour and 5 hours after brain death (BD) with placebo-pretreatment (BD group, in red) or with preventive methylprednisolone (BD + Corticosteroids group in blue). Values are expressed as mean ± SEM. * p<0.05 BD versus BD + Corticosteroids; † p<0.05 Base versus BD + 1 hour, ‡ p<0.05 BD + 1 hour versus BD + 5 hours, § p<0.05 Base versus BD + 5 hours in the BD group; || p<0.05 Base versus BD + 1 hour, # p<0.05 BD + 1 hour versus BD + 5 hours, ** p<0.05 Base versus BD + 5 hours in the BD + Corticosteroids group.

In the early phase (BD + 1h), brain death was associated with increased, PAP, pulmonary vascular resistance (PVR), PCP and venous component of PVR (Rv), HR, CO, noradrenaline need (used to maintain stable SAP), RAP and a decreased PaO_2_/FiO_2_ (Figs 1 and 2).

In the late phase (BD + 5h), brain death was associated with further increases in PVR, noradrenaline need, and with further decrease in PaO_2_/FiO_2_, with no further changes in HR, RAP, mean PAP, Rv, PCP, HR and RAP (Figs 1 and 2).

Brain death was associated, with no change in SAP (maintained with significant and progressive increase in noradrenaline need) and in PAWP (Figs 1 and 2).

#### Brain death after methylprednisolone pretreatment

Pretreatment with methylprednisolone did not prevent hemodynamic changes induced one hour after brain death (Figs 1 and 2).

Five hours after Cushing reflex, methylprednisolone pretreatment prevented brain death-induced changes in PAP, PVR, PCP, HR, CO and noradrenaline need, and reduced changes in PaO_2_/FiO_2_ (Figs 1 and 2).

#### Correlations

There was a significant correlation between PCP and PaO_2_/FiO_2_ (R = -0.4862; p<0.001) and between Rv and PaO_2_/FiO_2_ (R = -0.3358; p<0.05) (Fig 1).

### Evaluation of acute lung injury (ALI)

#### Brain death

**Fig 3:**
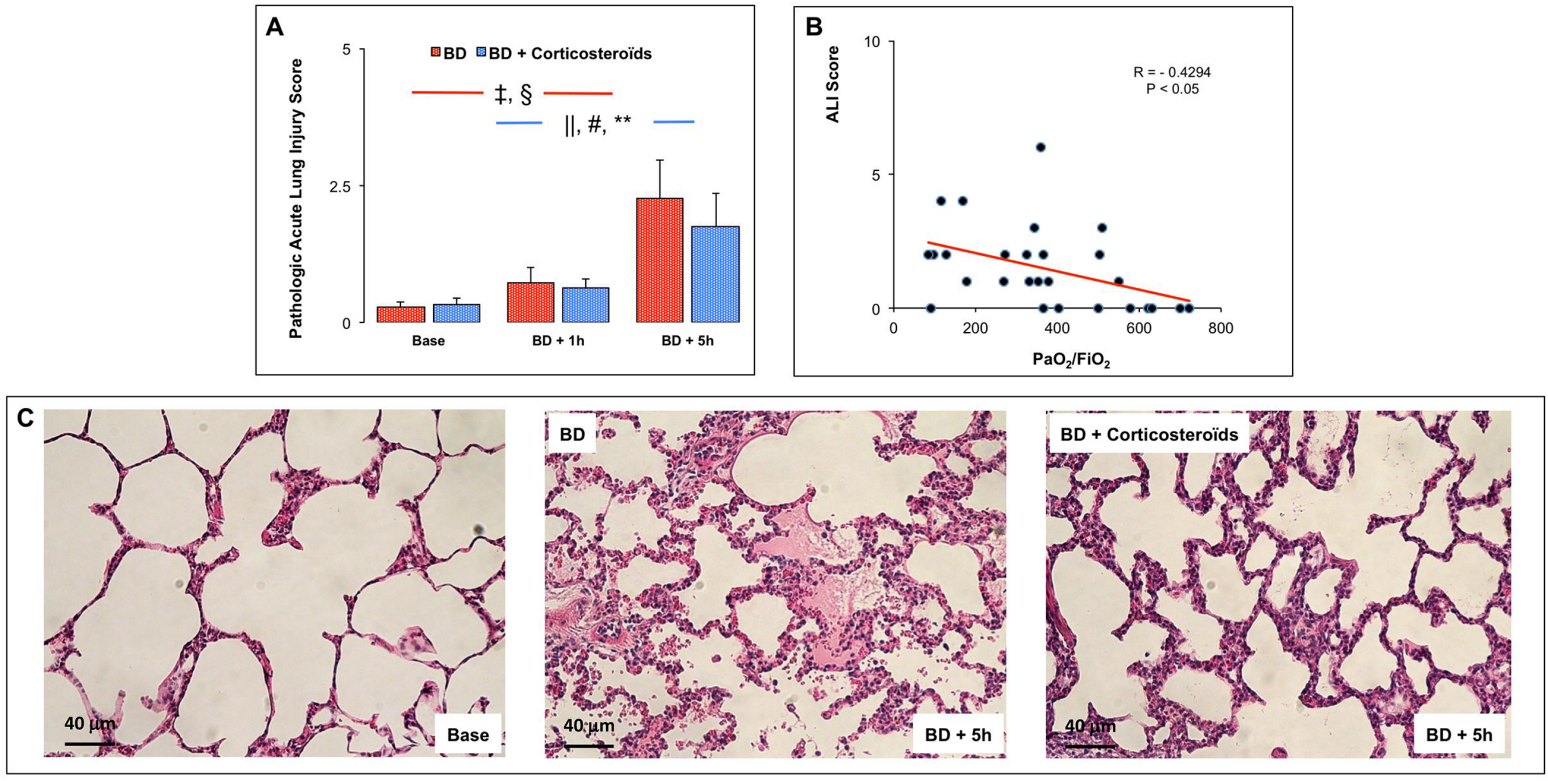
**Characterization of acute lung injury (Panel A)** at baseline, one (BD + 1 hour) and five (BD + 5 hours) hours after Cushing reflex in the placebo-pretreated brain death (BD group; n = 11; red bars) and the methylprednisolone-pretreated brain death (BD + Corticosteroids; n = 8; blue bars) groups. **Panel B:** Correlations between the ratio PaO_2_/FiO_2_ and ALI score. **Panel C:** Illustrative microscopic views (magnitude 200x) of hematoxylin-eosin stained lung sections at baseline and at 5 hours after Cushing reflex in the BD and the BD+Corticosteroids treated pigs. Values are expressed as mean ± SEM., ‡ p<0.05 BD + 1 hour versus BD + 5 hours, § p<0.05 Base versus BD + 5 hours in the BD group; || p<0.05 Base versus BD + 1 hour, # p<0.05 BD + 1 hour versus BD + 5 hours, ** p<0.05 Base versus BD + 5 hours in the BD + Corticosteroids group.

In the early (BD+1h) and the late (BD+5h) phases after brain death, the ALI score was increased (Fig 3).

#### Brain death after methylprednisolone pretreatment

The increase in ALI Score was not prevented by methylprednisolone pretreatment (Fig 3).

#### Correlations

The ALI Score and PaO_2_/FiO_2_ were correlated (R = -0.4294; p<0.05) (Fig 3).

### Evaluation of lung infiltration by neutrophils—Anti-myeloperoxidase (MPO) immunostaining. Brain death

**Fig 4:**
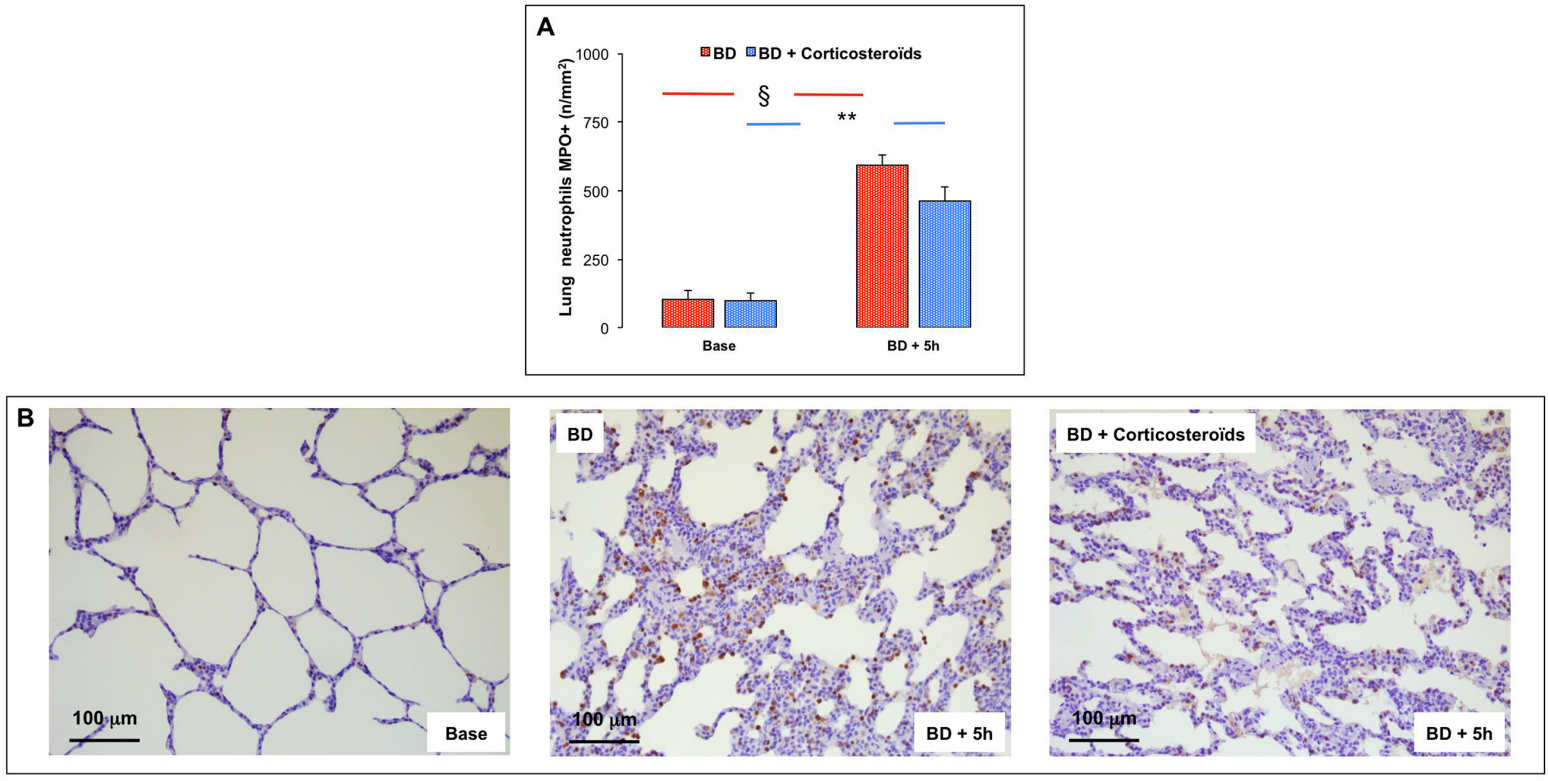
Evaluation of neutrophilic lung infiltration. **Panel A:** The number of extravascular neutrophils (MPO-positive cells) was determined in lung tissues at baseline, five (BD + 5 hours) hours after Cushing reflex in the placebo-pretreated brain death (BD group; n = 11; red bars) and in the methylprednisolone-pretreated brain death (BD + Corticosteroids; n = 8; blue bars) groups. **Panel B:** Illustrative microscopic views (magnitude 200x) of immunohistochemistry myeloperoxidase marked lung sections at baseline and at 5 hours after Cushing reflex in the BD and the BD + Corticosteroids pretreated pigs. Values are expressed as mean ± SEM. * p<0.05 BD versus BD + Corticosteroids; § p<0.05 Base versus BD + 5 hours in the BD group; ** p<0.05 Base versus BD + 5 hours in the BD + Corticosteroids group.

Brain death, in the late phase (BD + 5h), was associated with increased number of extravascular neutrophils per mm^2^ [assessed by immunohistochemistry (MPO)] (Fig 4).

#### Brain death after methylprednisolone pretreatment

The increase in extravascular neutrophils in lung tissue was not affected by methylprednisolone pretreatment (Fig 4).

### Lung expression of TNF-α and interleukins (e.g. IL-6, IL-10, IL-1β,IL-8)

#### Brain death

**Fig 5:**
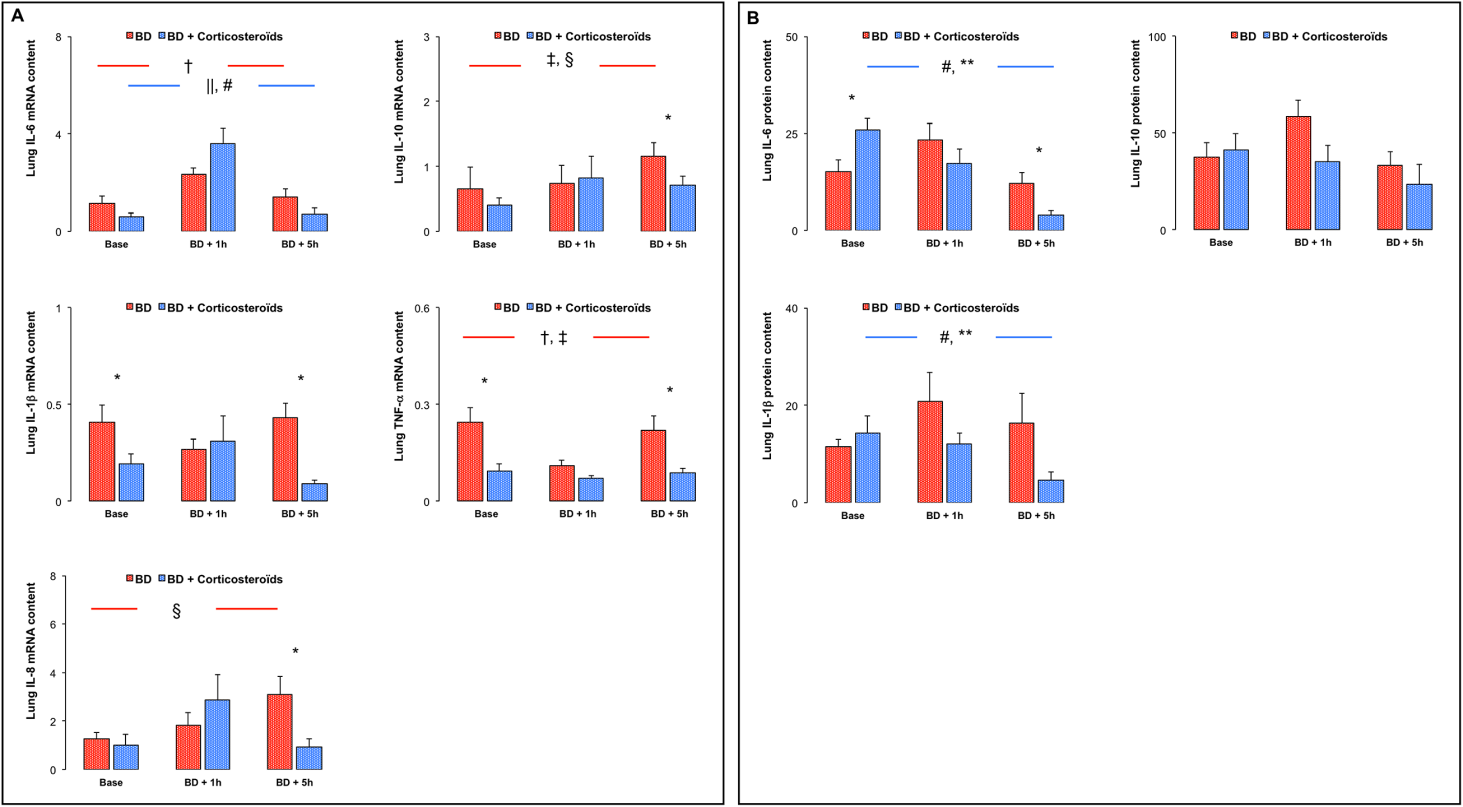
Lung expression of TNF-α and cytokines in brain death (BD)-induced lung injury. **Panel A.** Relative lung mRNA expression of interleukin (IL)-6, IL-10, IL-1β, TNF-α and IL-8 at baseline, one (BD + 1 hour) and five (BD + 5 hours) hours after Cushing reflex in the placebo-pretreated brain death (BD group; n = 11; red bars) and in the methylprednisolone-pretreated brain death (BD + Corticosteroids; n = 8; blue bars) groups. **Panel B:** Lung protein content for IL-6, IL-10 and IL-1β at baseline, one (BD + 1 hour) and five (BD + 5 hours) hours after Cushing reflex in the placebo-pretreated brain death (BD group; n = 11; red bars) and in the methylprednisolone-pretreated brain death (BD + Corticosteroids; n = 8; blue bars) groups. Values are expressed as mean ± SEM. * p<0.05 BD versus BD + Corticosteroids; † p<0.05 Base versus BD + 1 hour, ‡ p<0.05 BD + 1 hour versus BD + 5 hours, § p<0.05 Base versus BD + 5 hours in the BD group; || p<0.05 Base versus BD + 1 hour, # p<0.05 BD + 1 hour versus BD + 5 hours, ** p<0.05 Base versus BD + 5 hours in the BD + Corticosteroids group.

In the early phase (BD + 1h), brain death was associated with increased gene expression for IL-6, decreased TNF-α mRNA content with no changes in expression for IL-10, IL-1β and IL-8, resulting in increased pro-inflammatory IL-6 to IL-10 ratio (p<0.05) in total lung homogenates. There were no changes in lung protein content for IL-6, IL-10 and IL-1β (Fig 5).

In the late phase (BD + 5h), brain death was associated with further increased gene expression for IL-10 and IL-8, with no changes in IL-1β, IL-6, TNF-α and pro-inflammatory IL-6/IL-10 mRNA content in total lung homogenates. There were no changes in lung protein content for IL-6, IL-10 and IL-1β (Fig 5).

#### Brain death after methylprednisolone pretreatment

At baseline, methylprednisolone pretreatment was associated with decreased lung gene expression of IL-1β andTNF-α (Fig 5).

In the early phase (BD + 1h), methylprednisolone pretreatment was associated with no change in cytokines expression in total lung homogenates (Fig 5).

In the late phase (BD + 5h), methylprednisolone pretreatment was associated with decreased lung gene expression for IL-10, IL-1β, TNF-α, IL-8 and pro-inflammatory IL-6/IL-10 (p<0.05 compared to placebo-treated brain death). Corticosteroids pretreatment was associated with decreased lung protein content of IL-6 and IL-1β (trend) (Fig 5).

### Serum cytokine levels

#### Brain death

**Fig 6:**
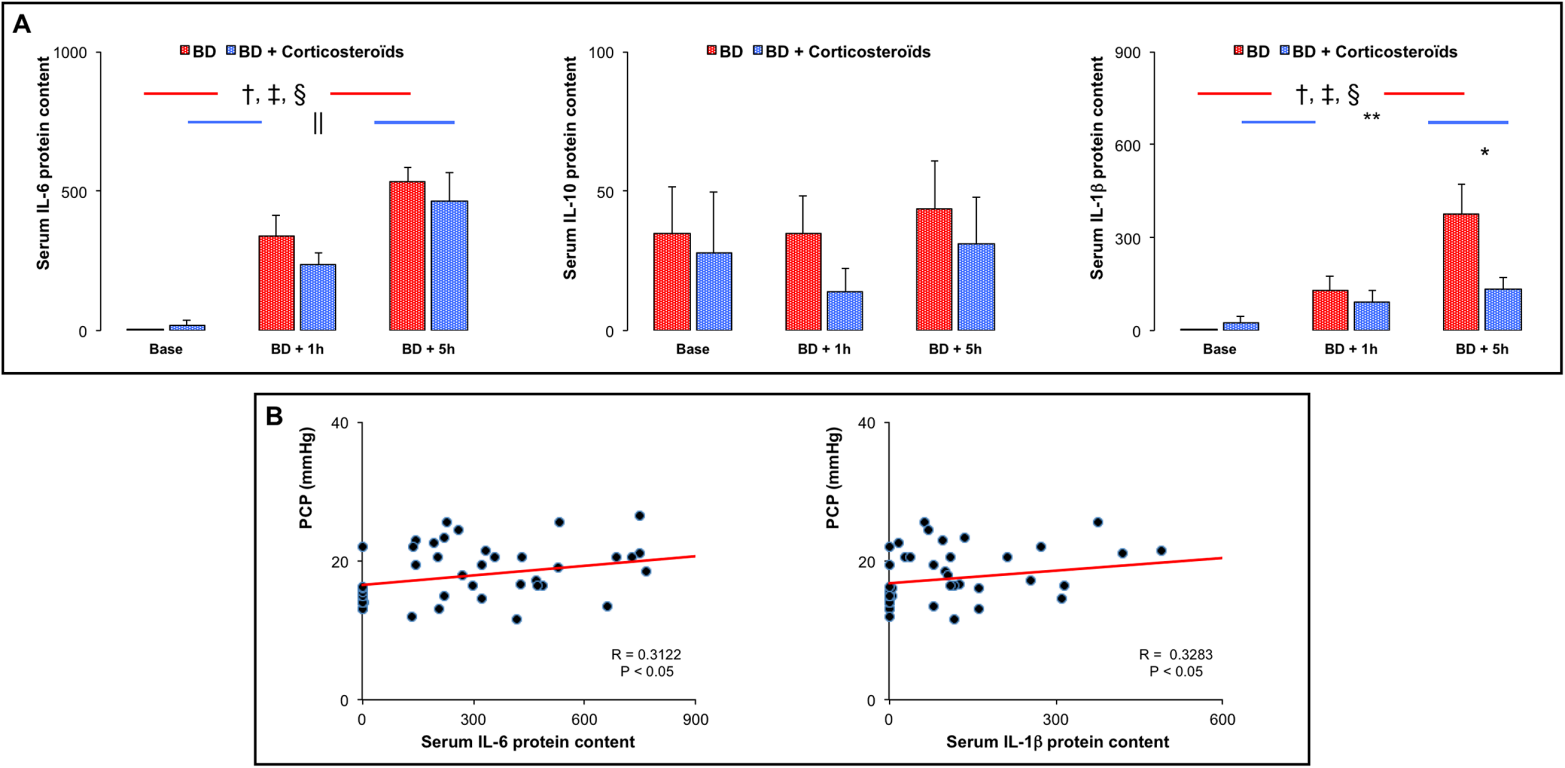
Serum interleukin (IL)-6, IL-10 and IL-1β protein concentrations. **Panel A:** Serum interleukin (IL)-6, IL-10 and IL-1β protein concentrations measurements at baseline, one (BD + 1 hour) and five (BD + 5 hours) hours after Cushing reflex in placebo-pretreated brain death (BD group; n = 11; red bars) and in the methylprednisolone-pretreated brain death (BD + Corticosteroids group; n = 8; blue bars) groups. Values are expressed as mean ± SEM. * p<0.05 BD versus BD + Corticosteroids; † p<0.05 Base versus BD + 1 hour, ‡ p<0.05 BD + 1 hour versus BD + 5 hours, § p<0.05 Base versus BD + 5 hours in the BD group; || p<0.05 Base versus BD + 1 hour, ** p<0.05 Base versus BD + 5 hours in the BD + Corticosteroids group. **Panel B:** Correlations between PCP and serum IL-6 and IL-1β.

Brain death, was associated with progressive increase in IL-6 and IL-1β serum concentration during both early (BD + 1h) and late (BD + 5h) phases of brain death. No change in serum IL-10 level was observed (Fig 6).

#### Brain death after methylprednisolone pretreatment

Methylprednisolone pretreatment prevented changes in IL-1 β serum concentration in the late phase of brain death but did not prevent changes in IL-6 serum concentrations (Fig 6).

#### Correlations

PCP was correlated to serum IL-6 (R = 0.3122; p<0.05) and IL-1β (R = 0.3283; p<0.05) concentrations (Fig 6).

### Lung expression of cell adhesion molecules (e.g. ICAM-1 and VCAM-1).

#### Brain death

**Fig 7:**
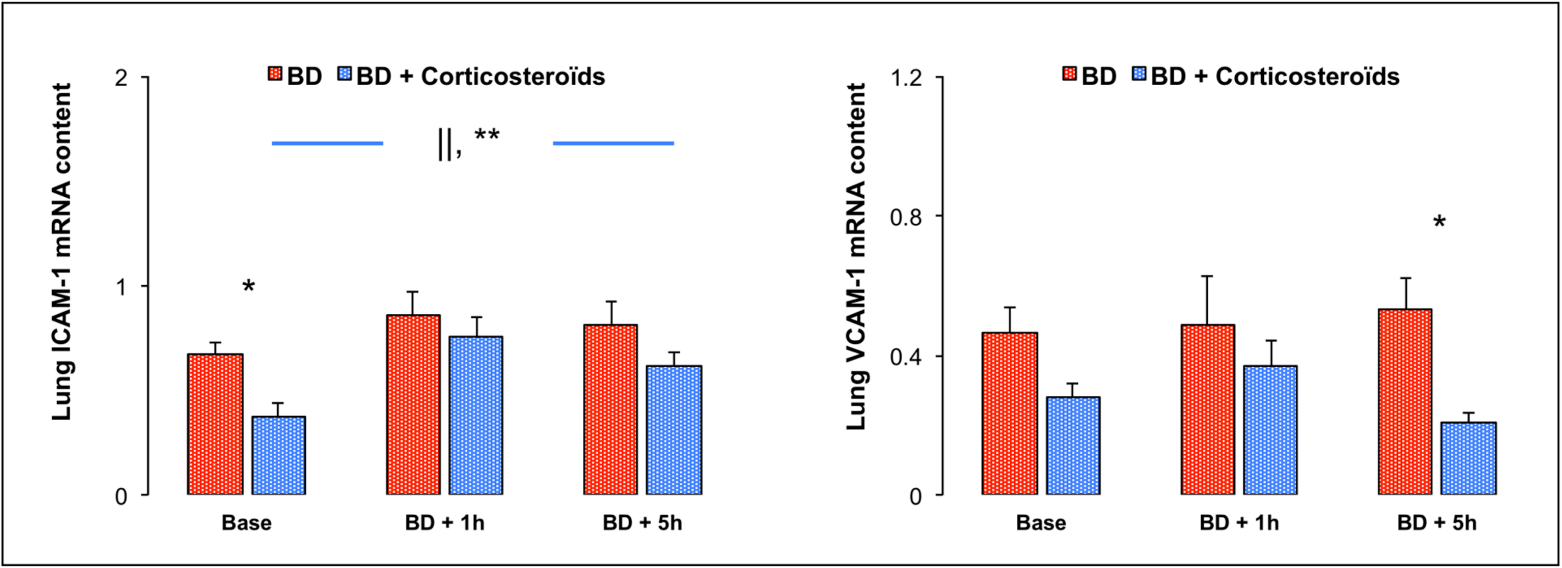
**Relative lung mRNA expression of intercellular adhesion molecule (ICAM) and vascular cell adhesion molecule (VCAM)-1** at baseline, one (BD + 1 hour) and five (BD + 5 hours) hours after Cushing reflex in the placebo-pretreated brain death (BD group; n = 11; red bars) and in the methylprednisolone-pretreated brain death (BD + Corticosteroids group; n = 8; blue bars) groups. Values are expressed as mean ± SEM. * p<0.05 BD versus BD + Corticosteroids; || p<0.05 Base versus BD + 1 hour, ** p<0.05 Base versus BD + 5 hours in the BD + Corticosteroids group.

At both early (BD + 1h) and late (BD + 5h) phases of brain death, lung gene expression for ICAM-1 and VCAM-1 were not changed (Fig 7).

#### Brain death after methylprednisolone pretreatment

In the late phase (BD + 5h), methylprednisolone pretreatment was associated with decreased lung VCAM-1 gene expression (Fig 7).

### Lung expression of oxidative stress molecules (e.g. HO-1, HIF-1α, GPx-1 and OXSR-1)

#### Brain death

**Fig 8:**
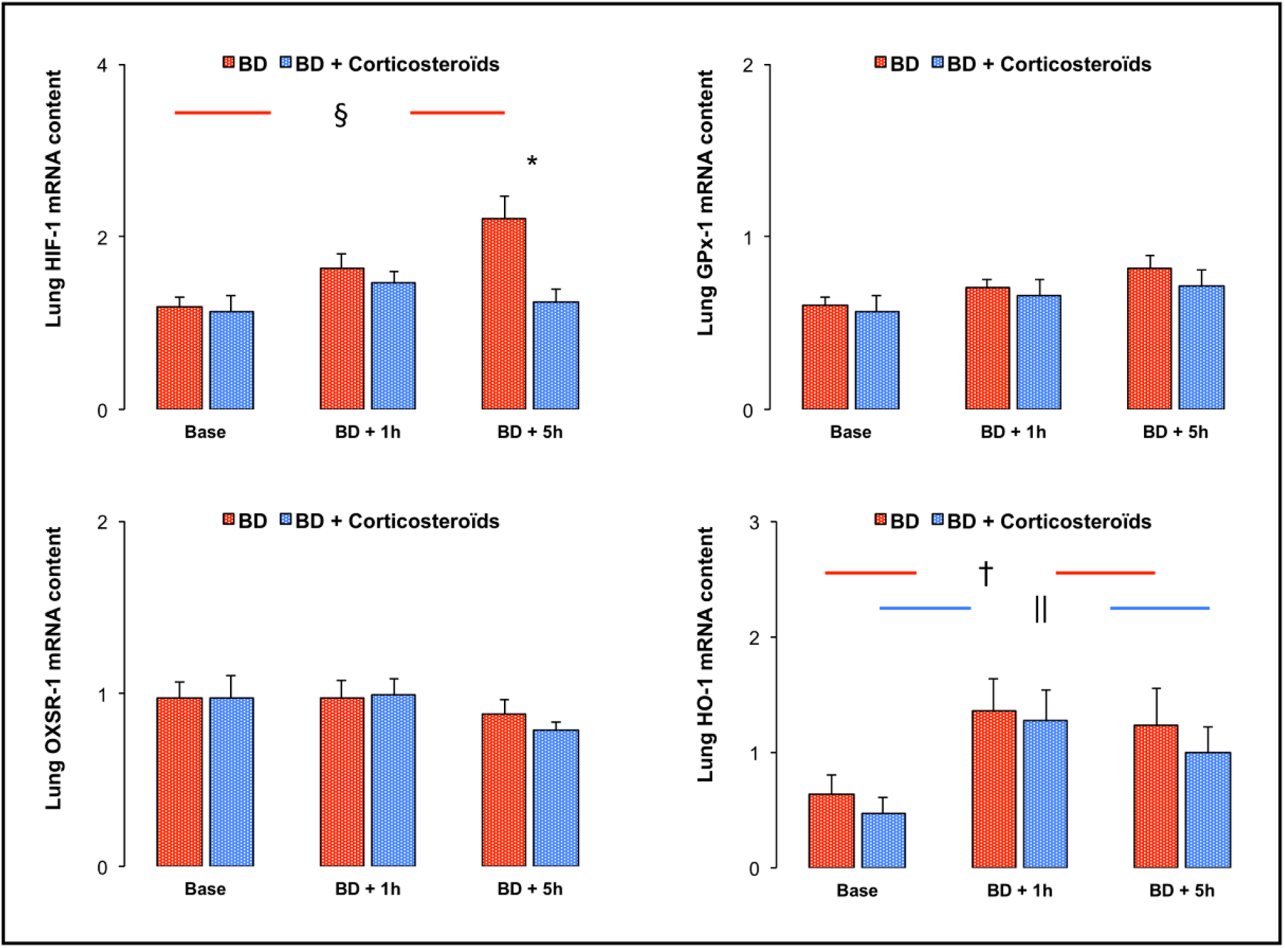
Oxidative-stress. Relative lung mRNA expression of Hypoxia inducible factor-1α (HIF-1α), Glutathion peroxidase-1 (GPx-1), Oxidative-stress responsive-1 (OXSR-1) and heme oxygenase-1 (HO-1) at baseline, one (BD + 1 hour) and five (BD + 5 hours) hours after Cushing reflex in the placebo-pretreated brain death (BD group; n = 11; red bars) and in the methylprednisolone-pretreated brain death (BD + Corticosteroids group; n = 8; blue bars) groups. Values are expressed as mean ± SEM. * p<0.05 BD versus BD + Corticosteroids; † p<0.05 Base versus BD + 1 hour, § p<0.05 Base versus BD + 5 hours in the BD group; || p<0.05 Base versus BD + 1 hour in the BD + Corticosteroids group.

Brain death was associated with increased lung expression for HO-1 in the early phase (BD + 1h) and with increased expression, five hours after Cushing reflex, for HIF-1α (Fig 8).

#### Brain death after methylprednisolone pretreatment

In the late phase of brain death (BD + 5h), methylprednisolone pretreatment prevented the increase in HIF-1α expression (Fig 8).

### Lung activation of apoptotic processes

#### Brain death

**Fig 9:**
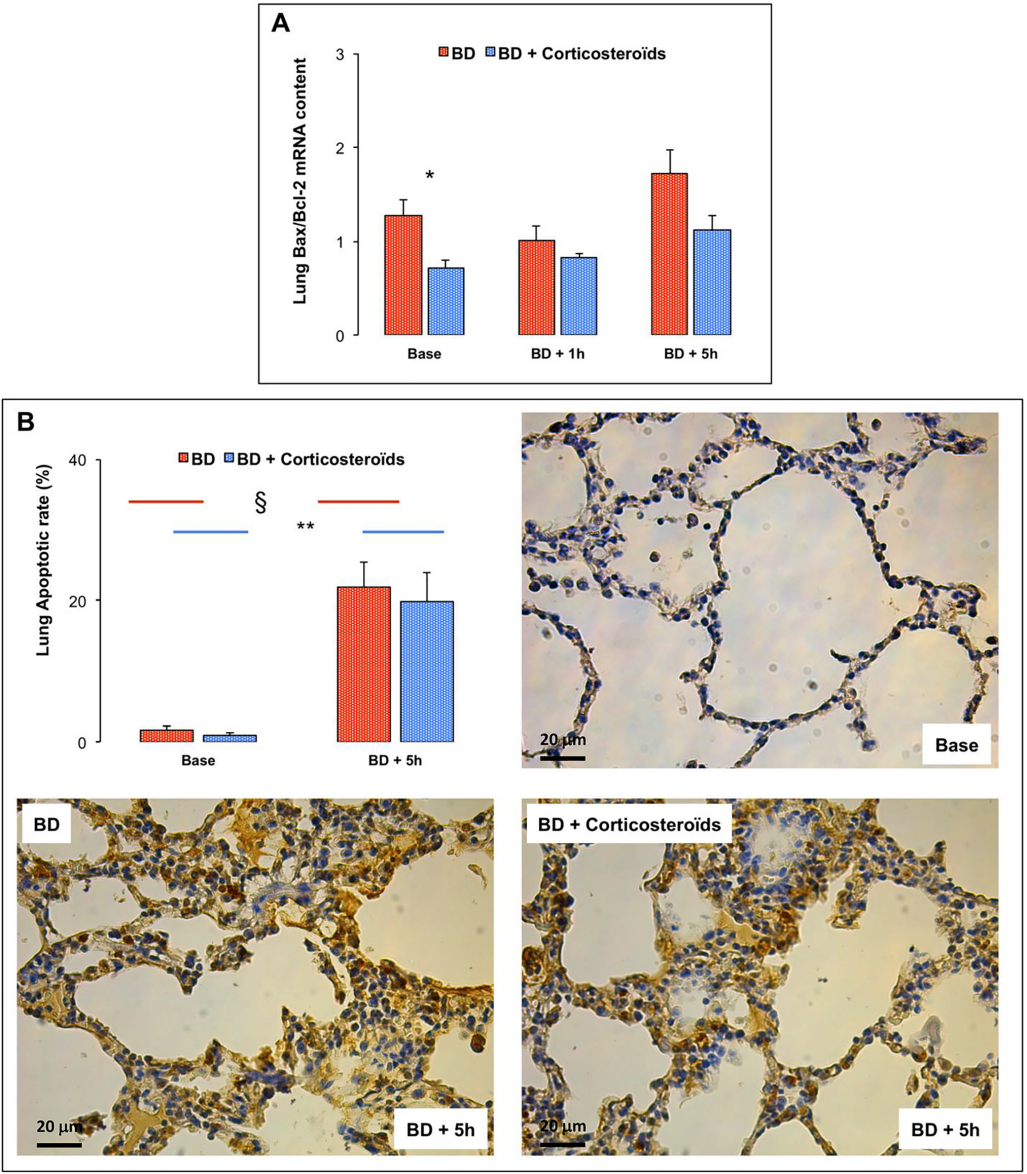
Lung apoptosis in brain death (BD)-induced lung injury with and without methylprednisolone pretreatment. **Panel A.** Pro-apoptotic Bax-to-Bcl-2 mRNA ratio at baseline, one (BD + 1 hour) and five (BD + 5 hours) hours after Cushing reflex in the placebo-pretreated brain death (BD group; n = 11; red bars) and in the methylprednisolone-pretreated brain death (BD + Corticosteroids group; n = 8; blue bars) groups. **Panel B.** Lung apoptotic rate (percentage) was evaluated as the ratio between the numbers of the terminal deoxynucleotidyl transferase biotin-dUTP nick-end labelling (TUNEL)-positive cells (brown nuclei) and the total number of cells (brown + blue nuclei) at baseline and five hours (BD + 5 h) after Cushing reflex in the placebo-pretreated brain death (BD group; n = 11; red bars) and in the methylprednisolone-pretreated brain death (BD + Corticosteroids group; n = 8; blue bars) groups. Illustrative microscopic TUNEL-immunostained lung sections (magnitude 400x) at baseline and at 5 hours after Cushing reflex in the BD and the BD + Corticosteroids treated pigs. Values are expressed as mean ± SEM. * p<0.05 BD versus BD + Corticosteroids; § p<0.05 Base versus BD + 5 hours in the BD group; ** p<0.05 Base versus BD + 5 hours in the BD + Corticosteroids group.

Brain death was associated with no changes in the Bax/Bcl2 pro-apoptotic ratio, but to assess the execution and the termination of apoptotic processes, TUNEL staining was performed. As illustrated in Fig 9B, in the late phase, brain death was associated with diffuse apoptosis in lung parenchyma (in extravascular areas) (Fig 9).

#### Brain death after methylprednisolone pretreatment

At baseline, the pro-apoptotic Bax/Bcl-2 ratio was decreased after methylprednisolone pretreatment. TUNEL staining changes were not prevented by methylprednisolone pretreatment (Fig 9).

### Multivariable analysis–ALI score and PaO_2_/FiO_2_

Multivariable analyses indicated PCP (R = -0.4767; p<0.05) and Rv (R = -0.5682; p<0.01) to be independent predictors of PaO_2_/FiO_2_ decrease at BD+1hr, and serum concentration of IL-6 to be independent factor (R = 0.6729; p<0.001) for PaO_2_/FiO_2_ decrease at BD + 5 hours.

In addition, multivariable analyses for ALI score indicated Rv (R = -0.5738; p<0.05) to be independent factor at BD + 1 hour, and lung gene expression for TNF-α (R = 0.5974; p<0.05) to be independent factor at BD + 5 hours.

## Discussion

For the first time, the present results show that porcine brain death (BD)-induced lung injury is associated with initial pulmonary capillary hypertension with increased pulmonary venous resistance. Brain death is also associated with altered gas exchange and expression or release of a number of pro-inflammatory cytokines which can be partially prevented by methylprednisolone therapy.

The pigs were of female sex because our animal facility is not equipped to accept more aggressive and difficult to handle males. This is admittedly a limitation of our study.

The Literature on experimental animal brain death models remains limited. The most widely used method relies in inflation of a Foley’s balloon in the subdural space. But this method does not reproduce the potential damage caused by intracranial blood effusion, which is most often present in clinical brain death.

Clinical and animal studies on subarachnoid hemorrhage report on an influx of immune cells associated with an increase in cytokine levels in both cerebrospinal fluid and serum [24]. In patients with subarachnoid hemorrhage, both ICAM-1 and VCAM-1 were increased in cerebrospinal fluid and serum [25,26]. An increase in IL-6, IL-1β and TNF-α was also detected in the serum of these patients [24,27]. Furthermore, lungs from brain death donors with cranial trauma are more frequently associated with early acute rejection and chronic bronchiolitis obliterans [28], which suggests an important physiological role for intracranial hemorrhage.

Based on these observations, it seemed interesting to develop a large animal model closer to human physiology when brain death is the consequence of intracranial hemorrhage. Until now, only one study described a model of intracranial hemorrhage [29] but without investigation of associated lung injury.

Even if results assumed that the brain death donor’s lungs may physiologically improve over time with progressive restoration of lung endothelial integrity [30] and rapid alveolar fluid resorption favorized by high levels of adrenal epinephrine and by diabetes insipidus installation [31,32], early hemodynamic damage and progressive systemic inflammatory response with massive increase in serum cytokine levels [7] could have an crucial role on the severity and control of pulmonary reperfusion injury. We therefore choose to study and identify the primary initial pathophysiological and biological mechanisms implicated in this condition.

In the present study, brain death-induced lung injury was associated with moderate pulmonary hypertension, as mean PAP exceeded the diagnostic cut-off value of 25 mmHg by only 2 mmHg, but with an increase in PCP above the threshold of 20 mmHg known to determine the onset of hydrostatic lung edema [32,33]. Pro-inflammatory Il-6/Il-10 were increased with the first measurement of increased PCP, allowing for reasonable speculation that lung injury at this stage was already caused by both increased PCP and capillary permeability.

Cerebrovascular stroke is the first cause of death in organ donors [34]. Intracranial accumulation of blood and associated cerebral edema may be a cause of rapid increase in intra-cranial pressure and activates a Cushing reflex to preserve cerebral perfusion [16] as was reproduced in the present experiments. This has been shown to be associated with a huge sympathetic nervous system activation, which may increase PCP because of associate blood volume shift from the systemic to pulmonary circulations, left heart failure of severe hypertension and catecholamine-induced myocarditis, and pulmonary venous constriction [1,2,6,35]. The present study did not attempt to capture Cushing reflex-associated events, but focused specifically on lung injury after established brain death. At this stage, CO, PAWP, stroke volume were within normal limits and SAP was low, excluding left heart failure on myocarditis of increased afterload. Yet PCP was abnormally high, in the range > 20 mmHg known to be associated with hydrostatic lung edema [32,33]. A possible explanation for this finding is persistent catecholamine-induced increased pulmonary venous resistance, previously reported as “pulmonary venule adrenergic hypersensitivity” [2]. Similar observations have been reported in high-altitude pulmonary edema (HAPE), where hypoxia and cerebral edema concur to hypoxic pulmonary vasoconstriction including the pulmonary venous segment to increase PCP [36]. In this particular condition too, high dose corticosteroids prevent symptoms of cerebral edema as well as increased pulmonary vascular pressures and lung injury [37].

In this porcine model of brain death-induced lung injury as well as in HAPE [38], initial stress on the pulmonary capillaries possibly due to transiently increased filling pressures of the left heart and more persistent venous constriction is soon followed by an inflammatory reaction which increases capillary permeability [39–41]. Broncho-alveolar lavage studies have reported on high pro-inflammatory cytokine concentration in broncho-alveolar lavage and serum and by increased polymorphonuclear cells and macrophages in the lungs after brain death [42,43]. This inflammatory response may be associated with an increased risk of chronic rejection after lung transplantation [12,43]. In the present study, we found early histologic signs of lung injury (evaluated as the acute lung injury score) with congestion, hemorrhage, and infiltration by neutrophils after brain death. This was accompanied by increased early lung expression of IL-6 and pro-inflammatory IL-6/IL-10, as well as by increased serum levels of IL-6 and IL-1β. This is consistent with previous studies suggesting that, these pro-inflammatory cytokines play a significant role in perpetuating lung injury [42,44,45] and supported by the results of multivariate analysis applied for ALI-score and PaO_2_/FiO_2_. IL-6, which is produced by many different cell types, such as macrophages, T- and B-lymphocytes and endothelial cells [46], is a pleiotropic cytokine influencing antigen-specific immune responses and inflammatory reactions. Although IL-1β and TNF-α are physiologic stimuli for the synthesis of IL-6, we failed to find altered expression for them after brain death. Several studies have shown elevated lung TNF-α and IL-1β expression [42,47,48] or no significant change in lung tissue TNF-α and IL-1β expression [49], suggesting variable expression of these cytokines after brain death. Lung tissue expression in IL-6 and pro-inflammatory IL-6/IL-10 returned to normal level five hours after brain death, suggesting their implication as an early inflammatory response to hemodynamic stress.

Transcription of intercellular adhesion molecule-1 (ICAM-1) and vascular cell adhesion molecule-1 (VCAM-1) is induced by TNF-α and IL-1β [50]. The expressions of ICAM-1 and VCAM-1 have been reported to be increased in toxic and BD-induced models of acute lung injury [48,49] but remained unchanged in the present study as reported by other authors [48]. The activation of apoptosis suggested by the increased lung apoptotic rate measured by TUNEL immunochemistry in the present study is also consistent with previous reports [51] and seems to be closely related to the activation of inflammatory processes.

Acute lung injury (ALI) and acute respiratory distress syndrome (ARDS) have been shown to be associated with an imbalance of cytokines, oxidative stress, and apoptosis [52]. TNF-α, IL-1β, and IL-6, have been shown to participate in the early development of inflammation, contacting and sequestrating neutrophils and lymphocytes that play crucial roles in the development and the maintenance of the conditions [53,54]. In a same way, in this study, we have measured lung tissue overexpression of IL-8 with an early imbalance in IL-6 to IL-10 ratio and an important lung tissue neutrophilic infiltration five hours after brain death.

Interestingly, IL-8 has been shown to be the predominant neutrophil chemokine present in the distal airspaces of patients with ALI and is a predictor of mortality in these patients [55,56]; blocking IL-8 significantly attenuates lung injury caused by acid aspiration or ischemia-reperfusion injury [57]. On another hand, neutrophils retention in the pulmonary capillary bed could lead to pulmonary microcirculation for mechanical obstruction, and the neutrophils could release a large number of inflammatory factors and oxidative stress which may increase the lung injury [58].

A growing number of studies have found that excessive production of Reactive oxygen species (ROS) and reduction of antioxidant defense systems play important roles in the pathogenesis of ALI [59]. Imbalance of oxidation and antioxidation mechanisms due to neutrophil activation and accumulation in the lung may produce large amounts of ROS causing direct damage to alveolar epithelial cells and pulmonary vascular endothelial cells with blood barrier integrity destroying and increased permeability, leading to pulmonary edema. Moreover, ROS activate the expression of NF-κB, which is known to play the most crucial role in cell apoptosis [60].

Because oxidative stress in lung tissue has been also considered to be important contributors to the pathogenesis of ALI [61], in this study, we have used lung tissues gene expression in hypoxia inducible factor-1-alpha (HIF-1α), heme oxygenase-1 (HO-1), glutathion peroxidase-1 (GPx-1), oxidative-stress responsive-1 (OXSR-1) as indicators to explore the potential oxidative stress role in brain death-induced lung injury in our model.

Under hypoxic conditions, oxygen-sensing machinery activates a transcription factor known as hypoxia-inducible factor 1 (HIF-1). This factor switches on a series of genes participating in compensatory mechanisms that support cell survival in a potentially lethal microenvironment as heme-oxygenase (HO)-1 [62]. Both HO-1 and HIF-1α were increased, in this study, suggesting activation of protective pathways against oxidative stress in brain death-induced lung injury in our model associated with dramatically decreased PaO_2_/FiO_2_.

It is generally believed that donor resuscitation with corticosteroids improves the inflammatory response to brain death. In the present study, pre-treatment with methylprednisolone improved pulmonary hemodynamics and gas exchange in brain death animals. Likewise, preventive dexamethasone treatment prevented pulmonary hypertension and associated increase in PCP, improved blood gases and preserved susceptible individuals from the development of HAPE [37]. Methylprednisolone could prevent increases in PAP and PCP by a decrease in venous resistance as shown in oleic acid lung injury [63] in part in relation to a still incompletely understood sympatholytic effect [37]. At pathobiological level, methylprednisolone pretreatment decreased IL-6/IL-10, IL-1β, IL-8, TNF-α and vascular cell adhesion protein (VCAM)-1, as well as decreased serum IL-1β level. However, it did not prevent acute lung injury associated to brain death. It could be partially explained by the absence of effects of methylprednisolone pretreatment on the oxidative stress markers. The administration of methylprednisolone 60-min after brain death in donor rats has been shown to reduce inflammatory activity in transplanted lungs, but has no influence on parameters related to oxidative stress [64]. Methylprednisolone has been, reported to limit endothelial cell damage [65] and therefore to prevent increased lung vascular permeability in experimental endotoxemia [66]. This mechanism could contribute to improve PaO_2_/FiO_2_ in the present experimental model of brain death, as suggested by inverse correlation between PCP and PaO_2_/FiO_2_.

## Conclusions

**Fig 10:**
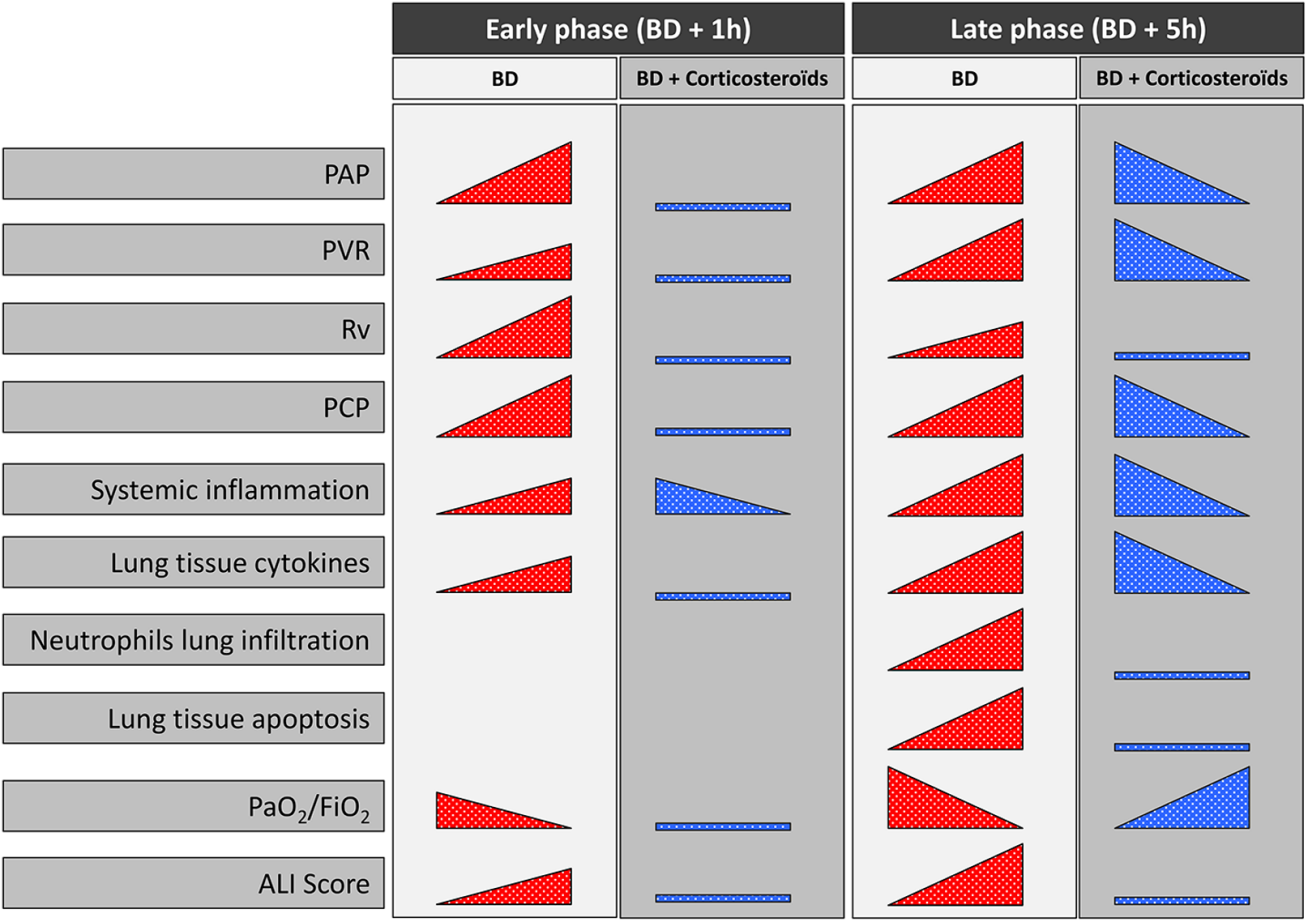
Findings summarize of the study. Brain death induced changes on hemodynamic evaluation [pulmonary artery pressure (PAP), pulmonary vascular resistance (PVR), pulmonary capillary pressure (PCP), venous compartmental resistance (Rv)], systemic inflammation, lung expression of cytokines, lung infiltration by neutrophils, PaO_2_/FiO_2_, and acute lung injury (ALI) Score at one (Early phase; BD + 1 hour) and five (Late phase; BD + 5 hours) hours after Cushing reflex in placebo-pretreated brain death (BD, in red) and in the methylprednisolone-pretreated brain death (BD + Corticosteroids, in blue) groups.

This study shows that brain death-induced lung injury is associated with initial pulmonary capillary hypertension with increased pulmonary venous resistance, what is shown for the first time, as a cause of altered gas exchange and expression or release of a number of pro-inflammatory cytokines which can partially prevented by vasodilating effects and anti-inflammatory actions of methylprednisolone therapy (Fig 10).

## Supporting information

S1 Data Datasets used and analyzed in the current study. Brain death (BD), heart rate (HR), cardiac output (CO), systemic arterial pressure (SAP), right atrial pressure (RAP), Noradrenaline Need, pulmonary artery wedge pressure (PAWP), pulmonary artery pressure (PAP), pulmonary vascular resistance (PVR), pulmonary capillary pressure (PCP), venous compartmental resistance (Rv), arterial PO_2_ divided by the fraction of inspired O_2_ (PaO_2_/FiO_2_), acute lung injury score (ALI Score), anti-myeloperoxidase immunostaining (MPO), interleukin 1β (IL-1β), interleukin 6 (IL-6), interleukin 10 (IL-10), interleukin 8 (IL-8), ribosomal protein L4 (RPL-4), TATA box binding protein (TBP-1), tumor necrosis factor alpha (TNF-α), Bcl2 associated X protein (Bax), B-cell Lymphoma-2 (Bcl-2), intercellular adhesion molecule 1 (ICAM-1), vascular cell adhesion molecule 1 (VCAM-1), heme oxygenase (HO-1), hypoxia inducible factor alpha (HIF-1α), Glutathion peroxidase 1 (GPx-1), Oxidative stress response 1 (OXSR-1).
(XLSX)

## Abbreviations

ACTB: Actin beta
ALI: Acute Lung Injury
Bax: Bcl2 associated X protein
Bcl-2: B-cell Lymphoma-2
BD: Brain death
CO: Cardiac output
CR: Cushing reflex
CS: Corticosteroids
ELISA: Enzyme-Linked ImmunoSorbent Assay
FiO_2_: Fraction of inspired oxygen
GPx: Glutathion peroxidase
HAPE: High-altitude pulmonary edema
HIF: Hypoxia inducible factor
HO-: Heme oxygenase
HPRT: Hypoxanthine phosphoribosyl transferase
HR: Heart rate
ICAM: Intercellular adhesion molecule
IL: Interleukin
IL-19: Interleukin 19
OXSR: Oxidative stress response
PaO_2_: Arterial pressure in Oxygen
PAP: Pulmonary artery pressure
PAWP: Wedge pulmonary artery pressure
Pcap: Pulmonary capillary pressure
PEEP: Positive end-expiratory pressure
PVR: Pulmonary vascular resistance
Q: Instantaneous pulmonary blood flow
RAP: Right arterial pressure
RPL4: Ribosomal protein L4
Rv: Venous component of pulmonary vascular resistance
SaO_2_: Arterial oxygen saturation
SAP: Systemic artery pressure
TBP1: TATA box binding protein
TNF-: Tumor Necrosis Factor
TUNEL: Terminal Deoxynucleotidyl Transferase dUTP Nick-End Labelling
VCAM: Vascular cell adhesion molecule
